# Synthesis and Herbicidal Activity of Substituted Pyrazole Isothiocyanates

**DOI:** 10.3390/molecules171012187

**Published:** 2012-10-17

**Authors:** Hua Wu, Jun-Tao Feng, Kai-Chun Lin, Xing Zhang

**Affiliations:** 1 Research & Development Center of Biorational Pesticide, Key Laboratory of Plant Protection Resources and Pest Management of Ministry of Education, Northwest A&F University, Yangling 712100, Shaanxi, China; 2 Shaanxi Research Center of Biopesticide Engineering & Technology, Northwest A&F University, Yangling 712100, Shaanxi, China; 3 Hubei Green-Tiandi Bioengineering Co., Ltd, Wuhan 430070, Hubei, China

**Keywords:** substituted pyrazoles, isothiocyanates, synthesis, herbicidal activity

## Abstract

Isothiocyanates and substituted pyrazoles were combined to form a series of novel isothiocyanates with highly effective herbicidal activity. The target compounds were analyzed by elemental analysis, ^1^H-NMR, EI-MS and IR spectroscopy. The synthesized compounds, particularly compounds **3**-**1** and **3**-**7**, exhibited good herbicidal activities against four weeds. The EC_50_ values of compound **3**-**1** against *Echinochloa crusgalli* L., *Cyperus iria* L., *Dactylis glomerata* L., and *Trifolium repens* L. were 64.32, 65.83, 62.42, and 67.72 µg/mL, respectively. The EC_50_ values of compound **3**-**7** against *E. crusgalli* L., *C. iria* L., *D. glomerata* L., *T. repens* L. were 65.33, 64.90, 59.41 and 67.41 µg/mL, respectively. Compounds **3**-**1** and **3**-**7** may be further optimized as lead compounds for new herbicides.

## 1. Introduction

The preparation and applications of isothiocyanates (ITCs) have been widely studied because they represent a class of important intermediates in organic synthesis. Isothiocyanates and their derivatives are extremely versatile. They can be used as pesticides and pharmaceutical intermediates to synthesize various types of heterocyclic compounds. Some isothiocyanate compounds can be directly applied as pesticides, while others can be used for the treatment of bacterial and inflammatory diseases and some cancers. Isothiocyanate compounds primarily prepared by chemists [1,2,3] and biologists [4,5] have gained widespread attention.

Recent studies have shown that some isothiocyanate compounds, specifically allylisothiocyanate (AITC) from the horseradish plant, exhibit good fumigation activities against storage pests, pathogenic microorganisms and root-knot nematode pests. Isothiocyanate compounds also inhibit seed germination in some weeds. For example, aromatic ITCs can inhibit seed germination in weeds such as *Matricaria inodora*, *Amaranthus hybridus* and *Echinochloa crusgalli* [6]. Norsworthy [7] demonstrated that tissues from cabbage and rape plants can effectively control *Vigna unguiculata* crop weeds. Haramoto and Gallandt [8] have reviewed the effect of substituted isothiocyanates against the seed germination of different weeds.

**Scheme 1:**
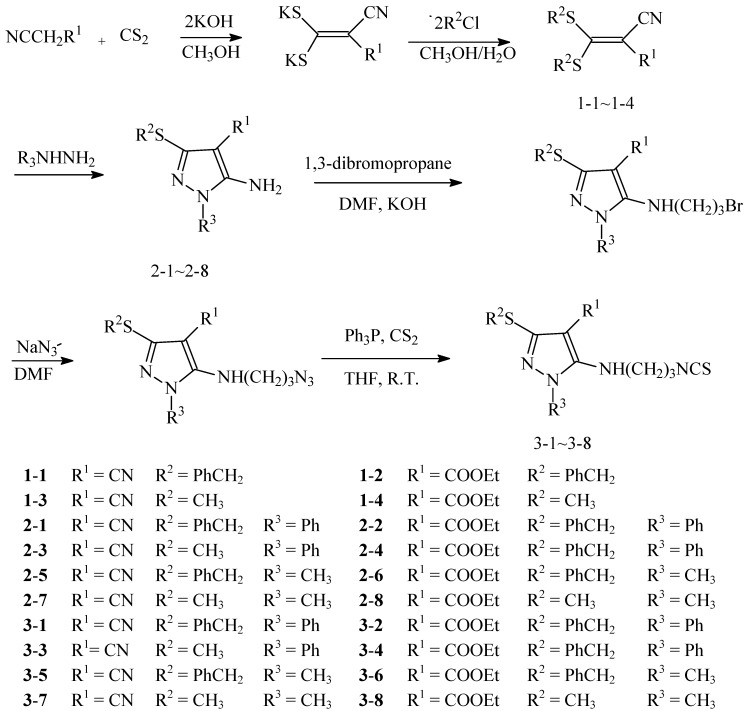
The synthesis route of compounds **1**-**1** to **1**-**4**, **2**-**1** to **2**-**8**, and **3**-**1** to **3**-**8**.

Pyrazole derivatives with agricultural activity have been patented and widely reported in previous studies [9,10,11]. Some pyrazole derivatives are used in commercial products, such as tebufenpyrad [12]. Studies on pyrazole amides have focused on 1-sulfonyl [13,14], aminoacyl [15], 5-benzylamide [12], and 4-amidopyrazoles [16]. Experiments on aryl pyrazole insecticide began in 1985 [17,18], and led to the successful development of Regent, a widely used insecticide [19,20]. There are some herbicides such as pyraflufenethyl (Japan, Idametsu), JV485 (Monsanto Bayer), which structure include pyrazole rings. Isothiocyanate compounds were also used to inhibit seed germination in some weeds. The isothiocyanate group could combine with the zymoproteins, which include sulfydryl amino acids (activation of apoptosis proteins), in the weeds, causing the weeds to die. According to the concept of isosteres, we combined isothiocyanates and substituted pyrazoles to produce a series of novel substituted pyrazole aminopropylisothiocyanates, seeking compounds with high herbicidal activity. The synthetic route is shown in Scheme 1.

## 2. Results and Discussion

### 2.1. The Properties of the Target Compounds

Eight target compounds were synthesized as white solids in six steps. The substitution reaction of amino-1-alkyl-3-alkylthio-5-aminopyrazole with 1,3-dibromopropane in DMF easily occurred in the presence of KOH. The reaction product was reacted with sodium azide. Hydrazoate compounds were isolated at the end of the reaction under anhydrous conditions; further reaction of the hydrazoate compounds with triphenylphosphine and carbon disulfide in THF produced the target compounds. The activity of the amino group at position 3 was high and reaction with dibromopropane occurred readily when the N at position 1 of the 1-alkyl-3-alkylthio-5-aminopyrazole ring was connected to a phenyl group. The activity of the amino group at site 3, as well as the target compound yield, decreased significantly when the N at position 1 was bonded to rejection electronic group of the methyl. The substituents at position 5 of the pyrazole ring exhibited minimal effects on the reactivity of the 3-amino site. The reaction conditions, particularly the reaction temperature, had a significant impact on the reaction yields. The synthesized compounds were characterized by ^1^H-NMR, IR, MS spectroscopy, and elemental analysis. ^1^H-NMR spectra of all of the compounds revealed was easily obtained at reasonable resolution. H shift values of the benzene ring compounds were within the expected range of δ = 7.0 to 7.6. A strong N=C=S absorption band between 2000 cm^−1^ to 2200 cm^−1^ was observed in the IR spectrum; a -CN stretching vibration absorption peak was found in the same range. The IR spectrum of compound **3**-**1** exhibited a broad absorption peak at 2098 cm^−1^ and 2222 cm^−1^, attributed to -NCS and -CN, respectively. A strong absorption peak was observed at 1500 cm^−1^ to 1604 cm^−1^; this peak was attributed to the C=C stretching vibrations. The MS spectra indicated that the synthesized compounds have strong molecular ion peaks.

### 2.2. Biological Activities

Results of the herbicidal activities of the tested compounds against the four weeds are listed in Table 1.

**Table 1:** Herbicidal activities of the samples against the four weeds *.

Compound	Corrected strain efficacy (%)			
	*Echinochloa crusgalli* L.	*Cyperus iria* L.	*Dactylis glomerata* L.	*Trifolium repens* L.
AITC (A)	25.86 ^f^	32.59 ^e^	40.12 ^e^	13.33 ^g^
**3**-**1**	79.39 ^b^	86.53 ^a^	79.26 ^a^	75.57 ^b^
**3**-**2**	65.32 ^c^	68.59 ^b^	64.53 ^b^	65.78 ^c^
**3**-**3**	54.62 ^d^	57.69 ^c^	55.62 ^c^	56.46 ^d^
**3**-**4**	58.93 ^d^	60.23 ^c^	59.08 ^b^	60.68 ^c^
**3**-**5**	44.62 ^e^	42.56 ^d^	31.25 ^f^	34.48 ^f^
**3**-**6**	63.32 ^c^	55.36 ^c^	46.53 ^d^	45.12 ^e^
**3**-**7**	78.69 ^b^	70.23 ^b^	74.33 ^a^	73.33 ^b^
**3**-**8**	52.32 ^d^	58.96 ^c^	56.25 ^c^	62.39 ^c^
chlorimuron ethyl ( **Z**)	98.32 ^a^	92.23 ^a^	78.25 ^a^	95.69 ^a^

Values are means of four replicates; Letters a, b, c, d, e, f and g represent a significant difference at *p* = 0.05. * The concentration of trial compounds and control herbicide were 100 μg/mL (75 g a.i./ha) and 20 μg/mL (15 a.i. g/ha), respectively. AITC (A): Allylisothiocyanate. **3**-**1**: 1-phenyl-3-benzylthio-4-cyanopyrazole-5-aminopropyl isothiocyanate; **3**-**2**: 1-phenyl-3-benzylthio-4-carbethoxy pyrazole-5-aminopropyl isothiocyanate; **3**-**3**: 1-phenyl-3-methylthio-4-cyanopyrazole-5-aminopropyl isothiocyanate; **3**-**4**: 1-phenyl-3-methylthio-4-carbethoxypyrazole-5-aminopropyl isothiocyanate; **3**-**5**: 1-methyl-3-benzylthio-4-cyanopyrazole-5-aminopropyl isothiocyanate; **3**-**6**: 1-methyl-3-benzylthio-4-carbethoxypyrazole-5-aminopropyl isothiocyanate; **3**-**7**: 1-methyl-3-methylthio-4-cyanopyrazole-5-aminopropyl isothiocyanate; **3**-**8**: 1-methyl-3-methylthio-4-carbethoxypyrazole-5-aminopropyl isothiocyanate.

Table 1 show that the tested compounds exhibited activity against the four kinds of weeds at a concentration of 100 mg/kg. The concentration of control herbicide (Z) was 20 μg/mL. The inhibitory activity sequence in *E. crusgalli* L. was Z > **3**-**1** > **3**-**7** > **3**-**2** > **3**-**6** > **3**-**4** > **3**-**3** > **3**-**8** > **3**-**5** > A, whereas in *C. iria* L., the inhibitory activity sequence was Z > **3**-**1** > **3**-**7** > **3**-**2** > **3**-**4** > **3**-**8** > **3**-**3** > **3**-**6** > **3**-**5** > A. The inhibitory activity sequence in *D. glomerata* L was **3**-**1** > Z > **3**-**7** > **3**-**2** > **3**-**4** > **3**-**8** > **3**-**3** > **3**-**6** >A > **3**-**5**; the inhibitory activity sequence in *T. repens* L. was Z > **3**-**1** > **3**-**7** > **3**-**2** > **3**-**8**> **3**-**4** > **3**-**3** > **3**-**6** > **3**-**5** > A. Significant differences between the compounds and the solvent control treatment were observed against the four weeds. Compounds **3**-**1** and **3**-**7** showed strong inhibitory activities against the four weeds at the tested concentration.

### 2.3. Rescreening Tests

Various concentrations (40, 60, 80, 100, and 120 µg/mL) of the synthesized compounds and (0.1, 1, 10, 20, and 30 µg/mL) controls were tested separately. Compounds **3**-**1** and **3**-**7** strongly suppressed the growth of weeds within the tested concentration range; compounds **3**-**1** and **3**-**7** distinctly suppressed the growth of weeds in a dose-dependent manner. The EC_50_ values of compound **3**-**1** against *E. crusgalli* L., *C. iria* L, *D. glomerata* L., and *T. repens* L. were 64.32, 65.83, 62.42, and 67.72 µg/mL, respectively. The EC_50_ values of compound **3**-**7** against *E. crusgalli* L., *C. iria* L., *D. glomerata* L., and *T. repens* L. were 65.33, 64.90, 59.41 and 67.41 µg/mL, respectively (Table 2). Compounds **3**-**1** and **3**-**7** may be further optimized as lead compounds for new herbicides.

**Table 2:** Growth suppression curve of compounds **3**-**1**, **3**-**7** and Z against four weeds.

Weeds	Samples	Correlation model	*R*	EC_50_ (µg/mL)
*E. crusgalli beauv* L.	**3**-**1**	Y = 3.29X−4.26	0.9907	64.32
	**3**-**7**	Y = 3.58X−5.07	0.9637	65.33
	Z	Y = 3.63X−2.88	0.9356	10.65
*C. iria* L.	**3**-**1**	Y = 2.89X−3.16	0.9976	65.83
	**3**-**7**	Y = 3.61X−5.16	0.9917	64.90
	Z	Y = 2.98X−6.42	0.9456	12.89
*D. glomerata* L.	**3**-**1**	Y = 2.45X−1.85	0.9895	62.42
	**3**-**7**	Y = 2.63X−2.31	0.9895	59.41
	Z	Y = 2.98X−5.33	0.9763	15.23
*T. repens* L.	**3**-**1**	Y = 2.52X−2.14	0.9850	67.72
	**3**-**7**	Y = 2.78X−2.87	0.9703	67.41
	Z	Y = 3.46X−6.39	0.9627	9.98

**3**-**1**: 1-phenyl-3-benzylthio-4-cyanopyrazole-5-aminopropyl isothiocyanate; **3**-**7**: 1-methyl-3-methylthio-4-cyanopyrazole-5-aminopropyl isothiocyanate; Z: chlorimuron ethyl.

## 3. Experimental

### 3.1. General

The melting points of the products were determined using an X6-type apparatus (Beijing Tech Instrument Co., Beijing, China), ^1^H-NMR spectra were obtained using a Bruker Avance 300 MHz spectrometer with CDCl_3_ as the solvent and TMS as the internal standard. MS spectra were obtained with an Agilent GC-MS (6890-5973i) instrument or Applied Biosystems Mariner API-TOF equipment. FTIR spectra were obtained using an AVATAR 330Thermo Nicolet FTIR spectrophotometer, and elemental analysis was conducted on a Vario EL III elemental analyzer.

### 3.2. Preparation of Intermediates

#### 3.2.1. Synthesis of Dithioacetals

A previously described method [21] was used to synthesize the dithioacetals (Scheme1). Compound **1**-**1** was prepared by the following method: potassium hydroxide powder (0.3 mol, 16.8 g) was added to a round-bottom flask (500 mL) containing acetonitrile (125 mL). The reaction mixture was cooled to 0 °C, and malononitrile (0.15 mol, 9.9 g) was added dropwise to it. The mixture was stirred at 10 °C, and carbon disulfide (0.15 mol, 11.4 g) was added dropwise to it. The mixture was stirred at room temperature for 2 h and then subjected to vacuum filtration. The filtered solids were washed with a small amount of ether, dried, and then transferred into a 250 mL round bottom flask containing 5:1 methanol-water (120 mL). The reaction mixture was stirred at (25 ± 2) °C while benzyl chloride (0.30 mol, 38 g) was added drpwise to it. A white solid precipitated from the solution. The mixture was stirred at 50 °C for 3 h until the reaction was completed. The reaction mixture was cooled in an ice bath, producing numerous light yellow solids that were precipitated rapidly, filtered, and washed with a small amount of ether. The resulting products were decanted and oven-dried for 8 h at 55 °C. Recrystallization was carried out using ethanol to obtain the pure compound. The pure compound **1**-**1** weighed 38.5 g (79.7% yield), and its melting point was 85.5 °C (lit. [22] 84–85 °C). Similar methods were used to prepare compound **1**-**2**, (81.2% yield), melting point 78 °C (lit. [22] 77–78.5 °C). Compounds **1**-**3** and **1**-**4** were also synthesized by similar methods. The pure compound **1**-**3** (23.8 g, 93.3% yield) featured a melting point of 82 °C (lit. [22] 80–81 °C). The pure compound **1**-**4** was obtained at 79.2% yield and had a melting point of 56 °C (lit. [22] 56–57 °C).

#### 3.2.2. Synthesis of 1-Alkyl-3-alkylthio-5-aminopyrazoles **2**-**1** to **2**-**8**

1-Phenyl-3-thiobenzyl-5-aminopyrazole-4-carbonitrile was prepared by the following method: a certain amount of 2-cyano-3,3-dithiobenzylacrylonitrile (0.005 mol, 1.61 g) was added to a round-bottom flask (250 mL) containing ethanol (30 mL). The flask was cooled to 0 °C. Phenyl hydrazine (0.005 mol, 0.54 g) in ethanol (10 mL) was added dropwise into the flask. The resulting solution was mixed, filtered, and heated to 80 °C to reflux for 6 h. The reaction mixture was cooled in an ice bath, and the solvent was removed by vacuum evaporation. The remaining solids were recrystallized from isopropanol to produce the pure compound **2**-**1**. Similar methods were used to prepare compounds **2**-**2** to **2**-**8** (Scheme 1).

#### 3.2.3. Synthesis of the Target Compounds **3**-**1** to **3**-**8**

Compound **3**-**1** was synthesized using the following method: compound **2**-**1** (0.01 mol, 0.306 g) was added to a round-bottom flask (250 mL) containing *N*,*N*-dimethylformamide (50 mL). The reaction flask was heated to 25 °C. Potassium hydroxide powder (0.01 mol, 0.56 g) and 1,3-dibromopropane (0.01 mol, 2.01 g) were added dropwise into the flask through a funnel in priority order under constant pressure. The solution was stirred at room temperature for 6 h until the reaction was completed and then filtered; the filtrate was concentrated under vacuum. The remaining solid was transferred to a 100 mL reaction flask with *N*,*N*-dimethylformamide (20 mL). Sodium azide (0.015 mol, 0.84 g) in *N*,*N*-dimethylformamide (10 mL) was added to the reaction flask, and the flask was heated to 45 °C for 3 h. The reaction mixture was filtered, and the filtrate was concentrated. The remaining solid was transferred into a 100 mL reaction flask containing tetrahydrofuran (30 mL). Triphenylphosphine (0.01 mol, 2.62 g) with tetrahydrofuran (20 mL) was added into the flask. The mixture was stirred at room temperature and carbon disulfide (0.015 mol, 1.14 g) of was added to it. The reaction mixture was stirred at room temperature for 6 h until the reaction was completed. The crude target products were obtained by vacuum concentration and filtration. The concentrated mixture was applied to a silica gel column and eluted using a mixture of petroleum ether, ethyl acetate, and dichloromethane (4:2:4, v/v/v) to produce compound **3**-**1**. Similar methods were used to synthesize compounds **3**-**2** to **3**-**8** (Scheme 1).

*1-Phenyl-3-benzylthio-4-cyanopyrazole-5-aminopropyl isothiocyanate* (**3**-**1**). White solid, m.p. 101–102 °C, ^1^H-NMR (CDCl_3_): δ = 2.24–2.28 (m, 2H, -CH_2_**CH_2_**CH_2_-), 3.91–3.92 (m, 4H, -**CH_2_**CH_2_**CH_2_**-), 4.28 (s, 2H, Ph**CH_2_**-), 7.24–7.45 (m, 10H, Ph); IR (KBr) ν/cm^−1^: 3068 (CH), 2967, 2876 (CH_2_), 2222 (CN), 2098 (NCS), 1723 (C=C), 1594, 1549, 1072, 927, 760, 699 (Ph); EI-MS: 405, 347; Anal. Calcd. For C_21_H_19_N_5_S_2_: C 62.20, H 4.72, N 17.27, S 15.81; Found: C 62.18, H 4.70, N 17.31, S 15.88.

*1-Phenyl-3-benzylthio-4-carbethoxypyrazole-5-aminopropyl isothiocyanate* (**3**-**2**). White solid, m.p*.* 85–86 °C, ^1^H-NMR (CDCl_3_): δ = 1.72–1.92 (t, 3H, -CH_2_**CH_3_**), 2.00–2.02 (m, 2H, -CH_2_**CH_2_**CH_2_-), 3.91–3.92 (m, 4H, -**CH_2_**CH_2_**CH_2_**-), 4.24–4.31 (m, 4H, Ph**CH_2_**-, -**CH_2_**CH_3_), 7.21–7.51 (m, 10H, Ph); IR (KBr) ν/cm^−1^: 3445 (N-), 3061, 3028 (CH), 2979, 2929 (CH_2_), 2097 (NCS), 1593, 1454, 1226, 1068, 1014,917, 786, 763, 695 (Ph); EI-MS: 453, 410; Anal. Calcd. For C_23_H_24_N_4_O_2_S_2_: C 61.04, H 5.34, N 12.38, O 7.07, S 14.17; Found: C 61.15, H 5.39, N 12.44, O 7.09, S14.15.

*1-Phenyl-3-methylthio-4-cyanopyrazole-5-aminopropyl isothiocyanate* (**3**-**3**). White solid, m.p. 112 °C, ^1^H-NMR (CDCl_3_): δ = 2.23–2.31 (m, 2H, -CH_2_**CH_2_**CH_2_-), 2.46–2.50 (s, 3H, -S**CH_3_**), 3.91–3.92 (m, 4H, -**CH_2_**CH_2_**CH_2_**-), 7.26–7.74 (m, 5H, Ph); IR (KBr) ν/cm^−1^: 3444 (N-H), 3054 (CH), 2926 (CH_2_), 2191 (CN), 2098 (NCS), 1433, 1103, 691, 638 (Ph); EI-MS: 329, 271; Anal. Calcd. For C_15_H_15_N_5_S_2_: C 54.69, H 4.59, N 21.26, S 19.46; Found: C 54.77, H 4.61, N 21.24, S 19.40.

*1-Phenyl-3-methylthio-4-carbethoxypyrazole-5-aminopropyl isothiocyanate* (**3**-**4**). White solid, m.p. 97 °C, ^1^H-NMR (CDCl_3_): δ = 1.36–1.39 (t, 3H, -CH_2_**CH_3_**), 2.08–2.16 (m, 2H, -CH_2_**CH_2_**CH_2_-), 2.46–2.50 (s, 3H, -S**CH_3_**), 3.82–3.86 (m, 4H, -**CH_2_**CH_2_**CH_2_**-), 4.27–4.29 (m, 2H, -**CH_2_**CH_3_), 7.26–7.74 (m, 5H, Ph); IR (KBr) ν/cm^−1^: 3055 (CH), 2977, 2882 (CH_2_), 2201 (CN), 2109 (NCS), 1596 (CO); 1224, 994, 930, 773, 704, 637 (Ph); EI-MS: 376, 318; Anal. Calcd. For C_17_H_20_N_4_O_2_S_2_: C 54.23, H 5.35, N 14.88, O 8.50, S 17.03; Found: C 54.25, H 5.37, N 14.90, O 8.51, S 17.05.

*1-Methyl-3-benzylthio-4-cyanopyrazole-5-aminopropyl isothiocyanate* (**3**-**5**). White solid, m.p. 85–86 °C, ^1^H-NMR (CDCl_3_): δ = 2.03–2.07 (m, 2H, -CH_2_**CH_2_**CH_2_-), 3.82 (s, 3H, -N-CH_3_), 3.93–3.95 (m, 4H, -**CH_2_**CH_2_**CH_2_**-), 4.28 (s, 2H, Ph**CH_2_**-), 7.20–7.53 (m, 5H, Ph); IR (KBr) ν/cm^−1^: 3028 (CH), 2943 (CH_2_), 2228 (CN), 2111 (NCS), 1716 (C=C), 1050, 699 (Ph); EI-MS: 343, 285; Anal. Calcd. For C_16_H_17_N_5_S_2_: C 55.95, H 4.99, N 20.39, S 18.67; Found: C 56.01, H 5.01, N20.35, S 18.65.

*1-Methyl-3-benzylthio-4-carbethoxypyrazole-5-aminopropyl isothiocyanate* (**3**-**6**). White solid, m.p. 92–93 °C, ^1^H-NMR (CDCl_3_): δ = 1.68–1.73 (t, 3H, -CH_2_**CH_3_**), 2.10–2.12 (m, 2H, -CH_2_**CH_2_**CH_2_-), 3.83 (s, 3H, -N-**CH_3_**), 3.88–3.92 (m, 4H, -**CH_2_**CH_2_**CH_2_**-), 4.24–4.28 (m, 4H, Ph**CH_2_**-; -**CH_2_**CH_3_), 7.24–7.62 (m, 5H, ph); IR (KBr) ν/cm^−1^: 3386 (N-H), 3061, 3028, 2985, 2933 (CH_2_), 2110 (NCS), 1632 (CO), 1048, 881, 689 (Ph); EI-MS: 390, 332; Anal. Calcd. For C_18_H_22_N_4_O_2_S_2_: C 55.36, H 5.68, N 14.35, O 8.19, S 16.42; Found: C 55.39, H 5.70, N 14.33, O 8.20, S 16.41.

*1-Methy-3-methylthio-4-cyanopyrazole-5-aminopropyl isothiocyanate* (**3**-**7**). White solid, m.p. 58–59 °C, ^1^H-NMR (CDCl_3_): δ = 2.00–2.02 (m, 2H, -CH_2_**CH_2_**CH_2_-), 2.46–2.50 (s, 3H, -S**CH_3_**), 3.91–3.92 (m, 4H, -**CH_2_**CH_2_**CH_2_**-), 3.84 (s, 3H, -N-**CH_3_**); IR (KBr) ν/cm^−1^: 3435 (N-H), 2967, 2940 (CH_2_), 2215 (CN), 2108 (NCS), 1499, 1062, 954, 711 (five-membered ring); EI-MS: 267, 209; Anal. Calcd. For C_10_H_13_N_5_S_2_: C 44.92, H 4.90, N 26.19, S 23.98; Found: C 45.01, H 4.92, N 26.15, S 24.00.

*1-Methyl-3-methylthio-4-carbethoxypyrazole-5-aminopropyl isothiocyanate* (**3**-**8**). White solid, m.p. 65–66 °C, ^1^H-NMR (CDCl_3_): δ = 1.72–1.92 (t, 3H, -CH_2_**CH_3_**), 2.01–2.02 (m, 2H, -**CH_2_**CH_3_), 2.00–2.02 (m, 2H, -CH_2_**CH_2_**CH_2_-), 2.46–2.50 (s, 3H, -S**CH_3_**), 3.82 (s, 3H, -N-**CH_3_**), 3.91–3.92 (m, 4H, -**CH_2_**CH_2_**CH_2_**-), 4.24–4.28 (m, 2H, -**CH_2_**CH_3_); IR (KBr) ν/cm^−1^: 3386 (N-H), 2965, 2933 (CH_2_), 2215 (CN), 2108 (NCS), 1590 (CO), 1065, 1019, 711, 636 (five-membered ring); EI-MS: 314, 256; Anal. Calcd. For C_12_H_18_N_4_O_2_S_2_: C 45.84, H 5.77, N 17.82, O 10.18, S 20.39; Found: C 45.80, H 5.79, N 17.81, O10.20, S 20.35.

### 3.3. Assay

#### 3.3.1. Test weeds

Seeds of weeds, including those of *Echinochloa crusgalli* L., *Cyperus iria* L., *Dactylis glomerata* L., and *Trifolium repens* L. were used. *E. crusgalli* seeds from the previous year were used. *C. iria*, *D. glomerata*, and *T. repens* were purchased from Wuhan Fu Yue Seed Industry Co., Ltd. (Wuhan, China).

#### 3.3.2. Experimental Treatments

Weed seeds were immersed in water for 48 h, and then incubated at 30 °C for 36 h until more than 80% of the seeds had germinated. Weeds were selected and transplanted to field plots when the growth of leaves was bent. Each plot contained 20 potted plants, and pots were placed in a greenhouse with a temperature of 25 °C to 28 °C, 60% to 80% humidity, and good light. The weeds that germinated were transferred to field plots, with each plot containing 20 strains of weeds. The stem and leaf spray method was used to test samples (100 μg/mL). Each treatment was repeated four times, with the same water (L), solvent controls (K) and the positive control [chlorimuron ethyl, (Z)].

#### 3.3.3. Rescreening Tests

Various concentrations of the synthesized compounds were applied to weeds determine the optimal concentration of compounds necessary to kill weeds.

#### 3.3.4. Survey Methods

After 14 d of spraying, live weeds were counted and the correction strain efficacy was calculated according to a previously described method [23].

#### 3.3.5. Statistical Analysis

Experimental data of herbicidal activity were analyzed using SPSS 16.0 for Windows.

## 4. Conclusions

In conclusion, a series of novel herbicides, in which the common pyrazole was substituted by isothiocyanates, were designed and synthesized. Most of the compounds exhibited excellent herbicidal activities against *Echinochloa crusgalli* L., *Cyperus iria* L., *Dactylis glomerata* L., and *Trifolium repens* L., which implied that the reaction of pyrazole with the electrophilic system to obtain novel isothiocyanate analogues with high activities was feasible. Compounds **3**-**1** and **3**-**7** may be further optimized as new herbicide lead compounds.
